# VERMONT: Visualizing mutations and their effects on protein physicochemical and topological property conservation

**DOI:** 10.1186/1753-6561-8-S2-S4

**Published:** 2014-08-28

**Authors:** Sabrina A Silveira, Alexandre V Fassio, Valdete M Gonçalves-Almeida, Elisa B de Lima, Yussif T Barcelos, Flávia F Aburjaile, Laerte M Rodrigues, Wagner Meira Jr, Raquel C de Melo-Minardi

**Affiliations:** 1 Department of Computer Science, Universidade Federal de Minas Gerais, Av. Antônio Carlos 6.627, 31270-901, Belo Horizonte, Brazil; 2 Department of Biochemistry and Immunology, Universidade Federal de Minas Gerais, Av. Antônio Carlos 6.627, 31270-901, Belo Horizonte, Brazil

**Keywords:** Mutation, Visualization, Molecular Structure and Function, Bioinformatics, Network Analysis

## Abstract

In this paper, we propose an interactive visualization called VERMONT which tackles the problem of visualizing mutations and infers their possible effects on the conservation of physicochemical and topological properties in protein families. More specifically, we visualize a set of structure-based sequence alignments and integrate several structural parameters that should aid biologists in gaining insight into possible consequences of mutations. VERMONT allowed us to identify patterns of position-specific properties as well as exceptions that may help predict whether specific mutations could damage protein function.

## Introduction

Some DNA mutations (i.e., substitutions, insertions and deletions), which naturally occur due to evolutionary pressure, are known to affect protein function. In fact, an important open problem in Bioinformatics is how specific modifications in protein amino acid properties may help identify potentially critical mutations, some of which may cause protein destabilization or significant structural modifications. Depending on where mutations take place, a protein may lose its function or become inactive.

A possible solution to this problem consists of collecting, integrating and processing a huge amount of information, ultimately presenting it in a simple manner in order to facilitate comprehension of the possible impacts of mutation. Our proposed solution involves two major premises: first, that protein structure data may give important clues about the impact of mutations on protein function and stability; and second, that a huge volume of different types of data must be integrated in order to improve the ability to study and predict harmful mutations. The resulting dataset is thus complex and difficult to analyze in a simple textual manner.

We believe that by presenting such data in a visualization which allows user interaction, much more interesting features, patterns, trends and exceptions may emerge from the dataset. Consequently, the goal of this work is to develop an interactive visualization tool that allows users to explore a diverse set of information about residues and mutations, enabling the identification of important mutations and their possible consequences on protein function.

The major contributions of this work are the investigation, selection and combination of a set of sequence-and structure-based data to estimate the impact of mutations, as well as the proposal of a visual representation of this multivariate dataset along with a selection of analytical interaction techniques that can boost analysis. Given that proteins may be modelled as networks of interacting atoms or residues, we mainly use as structure-based data protein topological features modelled as complex network measures, as well as physicochemical properties of interacting pairs.

The remainder of this paper is organized as follows: in *Methods*, we briefly describe the dataset, which has been previously detailed in this special issue, the types of supplementary data computed and combined into VERMONT, and the visual representations, as well as their resources and the strategies used to present information. In *Results and discussions*, we present and discuss the identified set of mutations which may potentially impact protein function and how they were selected. Finally, in *Conclusion*, we summarize VERMONT features, the set of selected mutations and present perspectives for future work.

## Methods

### Raw sequences and structures

The protein sequences were obtained from the organizers of the BioVis Contest (see *Acknowledgements*). They comprise a functionally defective triosephosphate isomerase (dTIM) and its *S. cerevisiae *parent (scTIM), both with 248 residues, as well as 1.4Mb of raw sequences of all other known triosephosphate isomerases (TIMs). The structure of scTIM was obtained from the Protein Data Bank (PDB).

The PDB was searched for each of the provided sequences and all corresponding structures were retrieved, resulting in the 133 enzyme set used in this work. Multiprot [1] was used to perform a structure-based sequence alignment of all sequences against chain A of the wild type PDB protein 2YPI, hereinafter called 2YPI:A. In VERMONT, each sequence is depicted as a row where columns represent equivalent positions in this alignment.

Retrieved PDB structures were improved using the PDBest package [2], a tool developed by our group to clean, filter and standardize data from PDB. The following procedures were applied in this work: chain separation, identical chain and structure removal, and exclusion of chains with missing atoms. In case of comparative models, we used the first one depicted in the PDB file.

### Computed data

Although sequence-based predictors have shown good performance, it has been previously demonstrated that prediction quality may be further improved by introducing features derived from three-dimensional protein structures [3]. In this work, we consider sequence conservation from a structural perspective by performing structure-based pairwise sequence alignments to allow all sequences in the dataset to be compared to a reference protein, in this case a mutant and defective enzyme. Also, we enrich the dataset with several structural features such as presence at the active site and solvent accessibility, as well as various types of data computed from the chemical interactions that a residue may establish with its structural neighbors. In this section, we detail each data type and discuss the intuition behind its usage for tackling the problem of predicting harmful mutations.

#### Active sites

The most obvious question one must answer when analyzing a mutation occurring in a member of an enzyme family is whether or not it affects the active site. Therefore, the first and simplest structural feature considered in this work is the presence of a mutation in a previously described active site. Thus, for each analyzed mutation, VERMONT users may verify whether it occurs on a known active site.

Active site data were retrieved from Catalytic Site Atlas (CSA) [4], a database documenting enzyme active sites and catalytic residues. CSA defines active site residues either based on bibliographic references or, more automatically, on remote homology computed by PSI-BLAST alignments.

#### Solvent accessibility

Globular proteins present a small fraction of their residues completely exposed to solvent molecules while the majority of residues are in the protein core. It is well known that hydrophobic residues tend to hide from solvent, forming hydrophobic cores inside the protein structure. These core residues also tend to be in close contact forming many interacting pairs, in a packing that tends to be very conserved in each protein family. Because of the contacts that residues inside a protein structure establish, we believe a mutation in the core of the structure tends to have much more impact on protein stability than one of a residue completely exposed on the surface. Hence, one of the structural features we computed is solvent accessibility, in an attempt to aid users in detecting mutations that are likely destabilizing.

Solvent accessibilities were computed by software NAccess, available in http://www.bioinf.manchester.ac.uk/naccess/, which implements the algorithm developed by Lee and Richards [5]: a probe of a given radius is rolled around the surface of the protein, and the accessible surface is defined as the path traced by the probe's center. Typically, the 1.4Å radius of a water molecule is used, so as to consider the solvent-accessible surface. Absolute accessibility is given in Å2. Since each amino acid residue presents distinct volume and surface area, we work with relative accessibilities, which express the accessible surface as a percentage of that observed in a Ala-X-Ala tripeptide (to mimic the extended conformation).

#### Chemical interactions

Analogous to the aforementioned packing patterns, when analyzing protein structures, one may observe many chemical interactions being established by a residue. Such contacts are essential to protein folding and stabilization. One may often find close residues interacting by the hydrophobic effect as well as via salt bridges and hydrogen bonds, both of which are dipole interactions. The set of interactions that a residue can establish with its surrounding pairs and the cumulative energy involved may be used as evidence of the residue's importance in protein folding and stabilization. Previous works [6-13] have shown that the interaction patterns established by residues are very conserved across each protein fold and, thus, may be used to understand protein function and interaction with other molecules.

We believe protein contacts are a very strong structural feature that may be used to predict the impact of mutations on protein structure and function. For example, the non-conservative substitution of a highly connected residue may disturb the previously established contacts, destabilizing the protein and impacting its function.

In this work, we use a cutoff-independent approach to geometrically compute amino acid interactions at the atomic level, which we then mapped to the residue level. For each protein, we used the CGAL software library [14] to build a Voronoi diagram followed by its Delaunay tessellation [15,16] and, using both distance and physicochemical properties described in [17], we classified contacts into one of the following types: charged attractive, charged repulsive, aromatic, hydrophobic or hydrogen bond. To avoid long edges representing illegitimate contacts, Gromacs [18] was used to solvate protein chains (as previously discussed in [19]).

#### Topological properties

A complex network is a graph with non-trivial topological features or, in other words, that does not occur at random. As we have recurrently discussed in previous works, proteins may be modelled as graphs where nodes are residues (or atoms) and edges represent chemical interactions that exist between residue pairs.

In [20], the authors used complex networks to study the role of an amino acid in both local and global structures, as well as to determine the extent to which disease-associated mutations and Single Amino acid Polymorphisms (SAPs) differ in terms of their interactions with other residues. They showed that mutations are likely disease-associated when they occur at a high centrality and/or high degree site in the network. A node's centrality measures its relative importance within the graph, while its degree represents the number of connections it is involved in.

In this work, we computed some of the most often employed complex network centrality measures using the iGraph package [21] from R software [22], namely:

• The *degree *k_i _of node *i *in a graph is the number of edges connected to it [23]. For an undirected graph containing *n *nodes, the degree may be written in terms of the adjacency matrix as $k_{i}=\sum_{j=1}^{n}A_{ij}$.

• The *betweenness *is a centrality measure that represents the extent to which a node lies on paths among other nodes. Mathematically, let $n_{st}^{i}$ be 1 if node *i *lies on the geodesic path from *s *to *t*, and 0 otherwise or if there is no such path (because s and t lie in different network components). Then, the betweenness centrality *xi *is given by $x_{i}=\sum_{st}n_{st}^{i}$. This definition considers separately the geodesic paths in either direction between each node pair. Since our network is undirected, this effectively counts each pair twice, so we compensate by dividing the result by 2. Nodes with high betweenness centrality may have considerable inuence within a network due to their control over information passing among others.

• The *closeness *centrality measure represents the average distance from a node to all other nodes. Suppose *d_ij _*is the length of a geodesic path from *i *to *j *(i.e., the number of edges along the path). Then, the mean geodesic distance from *i *to *j*, averaged over all vertices *j *in the network, is $l_{i}=\frac{1}{n}\sum_{j}d_{ij}$. This measure takes low values for nodes that are separated from others by only a short geodesic distance on average. Such nodes might have better access to information at other nodes or more direct influence on them.

### Visualization

In this section, we describe the proposed visualization, discuss some of the challenges posed by the data and the requirements visualizations were expected to meet, as well as some project decisions we have made. All visual representations were implemented in D3 [24].

The dataset was originally composed of a extensive set of sequences of about 250 residues. We computed several physicochemical and topological properties for each residue, which demanded a visualization capable of representing these properties as well as the sequences. Therefore, we have a multivariate quantitative visualization problem which requires a tool that allows domain specialists to compare multiple sequences and their parameters so as to reveal patterns and exceptions.

It is well known that random insertions, deletions and substitutions on the nucleotide sequence within a gene may change the amino acid sequence of the corresponding protein. Some mutations do not drastically alter the protein's structure, but others do, thus impairing the protein's ability to function. Therefore, alignment strategies must be able to properly compare protein sequences. Once such robust alignments are available, proper visualization techniques are required to make sense of the similarities and dissimilarities between the set of sequences.

**Figure 1:**
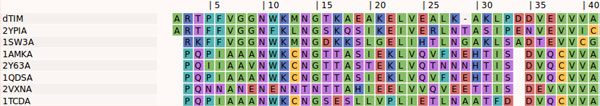
**Basis for the proposed visualizations**. Proposed visualization with CINEMA color scheme. Rows represent sequences and columns represent equivalent positions in the structure-based sequence alignment.

Biologists are used to visualizing and analyzing sequence alignments. Classical visualizations consist of depicting each sequence on a row, with columns representing equivalent positions in the alignment, where it is common to use color codes to aid the spotting of relevant conservations and exceptions in columns. Thus, an important requirement of the present visualization challenge was to stick to these traditional visual representations very easily interpreted by biologists. Consequently, the traditional visualization of multiple sequence alignments is the basis for those produced in this work and is shown in Figure 1. In addition to displaying aligned sequences, we include a varied set of physicochemical and topological parameters and some techniques for analytical interaction.

#### Screen

**Figure 2:**
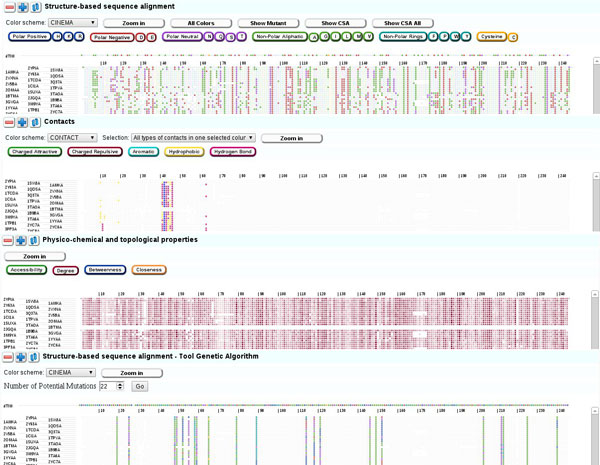
**VERMONT Screen**. Proposed visualization of the tool.

Figure 2 shows the screen of the proposed tool. Four sections exist and users can decide whether to expand or compress each one. There is also an option to see a reduced subset of the sequences using a scroll bar.

#### Structure-based sequence alignment

On the first section, we present a structure-based sequence alignment in which users may analyze the protein family in terms of sequence conservation throughout evolution. The mutant and the wild type proteins are presented in the first and second rows, respectively. In this panel, users may use three different color schemes, which were adapted from http://www.bioinformatics.nl/~berndb/aacolour.html:

• CINEMA: distinguishes among *polar positives *(H, K, R), *polar negatives *(D, E), *polar neutrals *(N, Q, S, T), *non-polar aliphatic *(A, G, I, L, M, V), *nonpolar rings *(F, P, W, Y) and *cysteine *residues (C);

• CLUSTAL: distinguishes among broader groups (G, P, S, T), (H, K, R), (F, W, Y) and (I, L, M, V);

• LESK: divides the residues into *small non-polar *(A, G, S, T), *hydrophobic *(C, F, I, L, M, P, V, W, Y), *polar *(H, N, Q), *negatively charged *(D, E) and *positively charged *(K, R).

**Figure 3:**
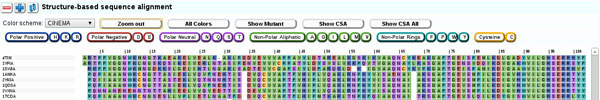
**Structure-based sequence alignment**. Proposed visualization for structure-based sequence alignment panel zoomed and filtered by polar residues.

Once a color scheme is selected, a set of selection buttons with the scheme groups is presented and users may filter residues individually or in groups by highlighting or attenuating them. Additionally, users may see the whole set of sequences in their full-length at a glance, as shown in Figure 2, or zoom in to see parts of the alignment in more detail, as depicted in Figure 3.

#### Contacts panel

The contacts panel works a bit differently from the others as it is intended to depict relationships between residues. Amino acid residues can interact with each other by establishing five types of chemical interactions: charged attractive, charged repulsive, aromatic, hydrophobic and hydrogen bond, which are distinguished by a color code. Three options are available for this panel:

• One type of contact in one selected column: in this case, users must click on a specific column and select a single type of contact to visualize with what other columns it interacts;

• All types of contacts in one selected column: users must click on a specific column to visualize all the established contacts, which are presented with an appropriate color scheme;

• One type of contact in all columns: this selection is a bit different and does not present contacts for a specific column, but for all the columns that establish the selected contact type.

#### Physicochemical and topological properties panel

This visualization combines the traditional sequence alignment visualization with a heatmap. It shows, using color intensities, the following measures: relative solvent accessibility, degree, betweenness and closeness. It helps users to spot conserved properties at specific alignment positions.

On all panels, users may obtain details on demand by passing the mouse over a residue cell. The tool will show residue type, position in the structure-based alignment and real position in the original sequence, as well as the values for all computed parameters.

The three panels were designed to help users visualize the whole set of sequences combined with the supplementary data added to support the process of selecting important mutations. The results presented at the BioVis Conference were obtained manually by experts and are presented in the *Results and Discussion *section. However, manual analysis is an arduous task considering the large number of sequences and amount of data to be evaluated. Therefore, we decided to develop a strategy to automatically select promising mutations by maximizing the number of different types of data that suggest probability of the mutation being deleterious, as explained in the following subsection.

### Automatic mutation selection

We developed a strategy based on Genetic Algorithms (GAs) [25-27] to automatically select mutations which could potentially lead to protein function damage. According to [28], GAs are stochastic search algorithms that act on a population of solutions and are inspired by the mechanics of population genetics and selection. Potential solutions are called \genes", which are strings of characters from a given alphabet. New solutions are generated by \mutating" members of the current population or by \mating" two solutions to form a new (hopefully better) one. Good solutions are selected to breed and/or mutate, while bad ones are discarded. Genetic algorithms are probabilistic search methods, which implies that the states they explore are not determined solely by the properties of the tackled problems. They are used in artificial intelligence to search a space of potential solutions for one which solves the problem at hand.

In the proposed GA, each possible solution to the problem is composed of a set of \genes", each of which correspond to a position (i.e., column) in the alignment provided in the *Structure-based sequence alignment *panel of VERMONT. The number of positions in a solution is a parameter that may be set by the user and can vary from 1 to 102, which is the total number of mutations in dTIM according to our alignment. To select the positions in which a mutation could potentially impact protein function, we proposed a *fitness *function that takes into consideration the same types of data used in the visualization. The main goal of the GA is to obtain a solution (i.e., a set of positions) which maximizes the *fitness*.

The *fitness *function takes into account *solvent accessibility, degree, betweenness, closeness, residue conservation *and *mutation score *of a column. These data are important because if residues with low solvent accessibility and high values of degree, betweenness and closeness are mutated, there is considerable chance that such a mutation impacts protein function. Also, it is important to consider the physicochemical features of residues involved in a mutation, seeing that non-conservative mutations (e.g., from Ala to Arg) have greater impact than conservative ones (e.g., from Ala to Gly). Therefore, and given that we compare similar sequences, substitution matrix PAM250 [29] was used to penalize non-conservative mutations. Other substitution matrices may be explored in the future. Equation 1 depicts the proposed *fitness *function, where N is the number of positions (i.e., columns) in a solution and Ti is the *partial fitness *for each position, defined by Equation 2

(1) $$Fitness\;=\overset{N}{\underset{i=0}{\sum }}T_{i}$$

(2) $$T_{i}=\left(1-Res_{freq}\right)+\left(1-Score\right)+\left(1-SRM\right)$$

Term *Res_freq _*corresponds to the frequency of the mutated residue in the column and *Score*, to the substitution matrix score, maximizing the partial fitness of non-conservative mutations. The SRM component is calculated by Equation 3, where *Acc_avg_, Deg_avg_, Bet_avg _*and *Clo_avg _*correspond, respectively, to the average accessibility, degree, betweenness and closeness normalized by the highest average of each parameter in the column. Because some parameters (e.g., accessibility) highly influence fitness when they have low values, we used 1 to equate the final result.

(3) $$SRM=\frac{ACC_{avg}}{ACC_{max}}+\frac{Deg_{avg}}{Deg_{max}}+\frac{Bet_{avg}}{Bet_{max}}+\frac{Clo_{avg}}{Clo_{max}}$$

The size of the GA population is set by parameter *population size*. During each step of the GA run, a population of individuals (i.e., possible solutions) is generated, so it is important to choose the promising ones to continue with in the next steps. In this work, such solutions were selected by tournament, a method in which a subset of K solutions is randomly picked from the population and, among them, the one with best *fitness *wins the tournament. Then, we apply to the selected solution(s) the genetic operators of *cloning, crossover *and *mutation*, detailed in [26]. Such operators are important to diversify the population while keeping the positive features acquired by previous generations.

The steps performed by the proposed GA are the following:

1 Randomly generate an initial population;

2 Calculate the fitness for all individuals in the current population;

3 Select two individuals in the current population by tournament and use them to generate two new individuals by performing *crossover*;

4 Repeat step 3 until a new population of same size is created composed of newly generated individuals and cloned individuals;

5 Apply the mutation operator to each individual of the new population;

6 Repeat steps 2 through 5 until the maximum number of generations is reached;

7 The solution will be the best individual in the last generation, i.e., the one with highest *fitness*.

It is important to point out that this GA is a work in progress which allows us to automatically choose mutations that may potentially change protein function by taking into consideration a variety of topological and physicochemical properties of residues. Although seemingly promising, as can be observed in the *Results and Discussions *section, we must carefully and deeply assess the importance of each property in the *fitness *function and their corresponding weights.

## Results and discussion

### Manually generated results

As previously mentioned, we decided to work only with sequences whose 3D structures were available, since structures are much more conserved than sequences and the vast majority of the supplementary data we used are derived from structures. Based on the alignment and analysis of the mutant sequence (dTIM) and its wild type protein (2YPI:A), we identified 102 mutations to further evaluate. Each single point mutation was studied from different perspectives: family residue conservation, as well as physicochemical and topological properties.

This work's main hypothesis is that important residues are conserved throughout evolution, so conserved positions in the structure-based sequence alignment are important for function preservation. As previously discussed, some mutations drastically alter a protein's structure and function. Some amino acid substitutions are commonly found throughout the molecular evolution process, while others are rare: Asn, Asp, Glu and Ser are the most mutable amino acids, while Cys and Trp are the least. It is important to mention that the substitution of an amino acid by another with similar physicochemical properties will likely not impact protein stability. Having this in mind, we classified amino acids according to three different schemes (i.e., CINEMA, CLUSTAL and LESK) and analyzed mutations as conservative or not.

Non-conservative mutations were prioritized in this work, seeing that they likely have greater impact on protein function. For each mutation, we manually verified if it was frequent, rare or very rare in the family. Frequent mutations probably do not impact function, since they occurred in other proteins of the family at a similar context and did not yield function loss. Therefore, only rare and very rare mutations were further investigated.

One of our first assessments when analyzing the BioVis Contest data was that every mutation was present in at least one other sequence of the family, which meant that no single mutation causing function loss was found. It is also important to mention that no mutations were found in protein active sites (N10, K12, H95, E165 and G171). Such mutations would obviously impact function. We have also investigated possible mutations in residues that are in contact with the active site, which could likely affect active site conformation and lead to function modification. However, no such mutation was found either.

**Table 1:** Predictions of harmful mutations: 22 mutations identified as possibly causing damage to protein function using our manual selection strategy.

Mutation	Avg. degree	Avg. betweenness	Avg. closeness (E-04)	Avg. accessibility
S19E	3.93	101.68	9.9	47.47
I20A	8.45	728.61	9.9	3.12
N28K	5.54	93.93	9.9	30.09
K56G	6.87	198.56	9.9	16.32
T60K	5.25	455.12	9.8	25.92
K69E	4.57	362.98	10.0	39.75
S71K	2.86	160.55	9.7	69.87
K89D	3.82	289.67	9.9	44.37
D111K	5.08	89.62	9.8	44.26
G118E	3.53	19.84	9.8	78.12
E152A	3.8	22.26	9.9	73.91
E153G	5.91	120.75	9.9	40.56
K155D	3.49	23.58	9.8	74.42
T158K	4.39	100.68	9.9	60.49
S194E	4.37	26.71	9.9	74.15
K195N	4.61	49.99	9.9	55.2
K199E	3.56	56.41	9.9	64.94
S202E	4.35	100.48	9.9	52.74
N213K	5.72	265.96	9.9	37.79
G214P	4.09	108.98	9.9	31.33
K221A	5.24	246.48	10.0	19.78
D222A	4.89	56.55	9.9	68.43

Sequence numbering according to PDB ID 2YPI:A

The non-conservative rare and very rare mutations were then carefully investigated regarding accessibility and topological properties, which lead to the set of significant mutations presented in Table 1. The 22 mutations we considered to be likely to cause problems in protein function were selected based on parameter values that reect the topological importance of the affected residues in the protein structure. Mutations I20A, K56G, T60K, K69E, K89D, E153G, N213K and K221A present the highest centrality values (i.e., degree, betweenness and closeness) and the lowest accessibility values, which indicates that the corresponding residues are buried in the hydrophobic core and consequently perform more atomic interactions. We observed many non-conservative mutations (S19E, N28K, S71K, G118E, E152A, T158K, S194E, K195N, S202E, G214P and D222A) that add or remove charged residues, which may cause the loss or gain of contacts. Finally, we also observed completely destabilizing non-conservative mutations such as D111K, K155D and K199E, which alter the physicochemical properties of the residue.

## Conclusion

In this paper, we propose VERMONT, an interactive tool to visualize mutations in the context of a protein family and infer their possible consequences on protein structure and function. We modelled the problem as that of spotting residue conservations together with the conservation of physicochemical and topological properties. The proposed interactive visualization provides a macro view of the structure-based sequence alignment as well as several other structural features.

The tool allows users to view, at a glance, a multivariate set of residue parameters, expanding or compressing panels and zooming out to see the full length of sequences or zooming in to focus on specific areas. Users may also filter residues individually or by groups of similar properties by highlighting or attenuating them, which aids visual spotting of patterns and exceptions.

Using the proposed visualization tool, we were able to predict 22 mutations we believe have a significant probability of causing damage to protein function, some of which seem to be more severe and have a high likelihood of causing function loss.

Finally, the development of a strategy to automatically select likely damaging mutations, thus aiding experts, is ongoing. It is based on the know-how developed by experts in this type of analysis and is inspired in genetic algorithms. It tries to find mutations with maximum probability of causing function damage using the data integrated in VERMONT. Table 2 shows the 22 mutations that can potentially cause damage to protein function according our automatic mutation selection based on GA.

**Table 2:** Predictions of harmful mutations: 22 mutations identified as possibly causing damage to protein function using our automatic GA-based strategy.

Mutation	Avg. degree	Avg. betweenness	Avg. closeness (E-04)	Avg. accessibility
I20A	8.45	728.61	8.2	3.12
R26A	6.76	236.21	7.69	33.79
Y49T	6.8	227.64	7.68	23.21
S50A	9.26	565.23	8.52	0.33
L53A	6.76	138.71	7.45	36.94
K56G	6.87	198.56	7.64	16.32
V59I	7.63	537.69	8.49	5.7
T60K	5.25	455.12	9.01	25.92
A66C	7.12	1016.93	9.32	3.35
V80P	9.2	719.95	8.77	1.19
W90Y	6.96	1275.57	10.24	6.37
F115H	6.48	416.05	8.57	22.09
Q119H	6.21	165.8	8.22	27.2
G122K	5.22	485.78	9.66	16.62
L147T	9.48	808.43	9.23	0.47
V150L	10.96	1109.65	9.48	0.67
E153G	5.91	120.75	7.98	40.56
L204V	9.26	1076.06	9.87	3.06
N213K	5.72	265.96	8.09	37.79
G214P	4.09	108.98	7.57	31.33
V226I	9.31	787.96	9.6	1.76
V241L	7.29	518.77	8.31	16.84

Sequence numbering according to PDB ID 2YPI:A

**Figure 4:**
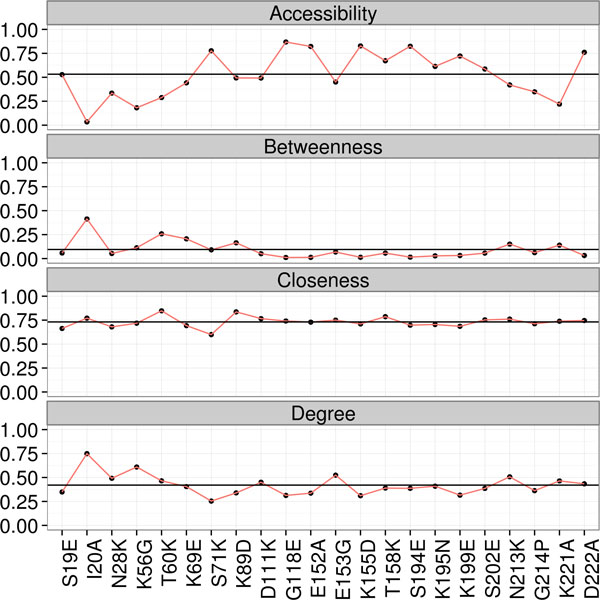
**Manually generated results**. Accessibility, betweenness, closeness and degree for manually generated results. Values from Table 1 were normalized by the highest value of the respective column.

**Figure 5:**
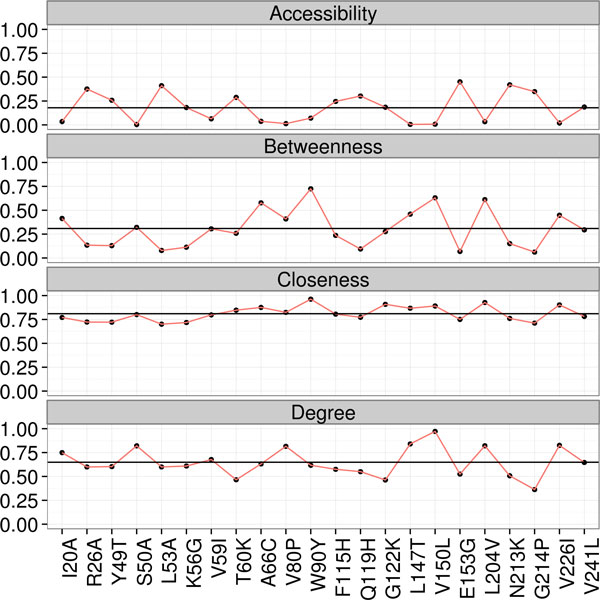
**Automatically generated results**. Accessibility, betweenness, closeness and degree for results generated using automatic GA-based strategy. Values from Table 2 were normalized by the highest value of the respective column.

Preliminary results comparing the manual and automatic strategies indicate that it is necessary to improve the fitness function or adjust the genetic algorithm parameters. Figures 4 and 5 show the mutant residues considered harmful for protein function, along with values of centrality measures for both strategies. A total of six mutations were found, namely I20A, K56G, T60K, E153G, N213K and G214P.

VERMONT is available in http://homepages.dcc.ufmg.br/~alexandrefassio/vermont/vermont.html.

## Competing interests

The authors declare that they have no competing interests.

## Authors' contributions

Yussif, Alexandre and Laerte implemented VERMONT's visual interface. Elisa, Sabrina and Valdete implemented the tools to compute the various measures used to make the predictions. Alexandre and Raquel designed the GA for performing automatic predictions, and Alexandre implemented and evaluated it. Sabrina, Raquel e Valdete analyzed the results and wrote the manuscript, while Elisa edited the revised manuscript. The other authors contributed to the discussion of the results. All authors reviewed the paper.

## Declarations

Publication costs were supported by the Brazilian agencies Coordenação de Aperfeiçoamento de Pessoal de Nível Superior (CAPES), Conselho Nacional de Desenvolvimento Científico e Tecnológico (CNPq), Fundação de Amparo à Pesquisa do Estado de Minas Gerais (FAPEMIG), Financiadora de Estudos e Projetos (FINEP) and Pró-Reitoria de Pesquisa of Universidade Federal de Minas Gerais.

This article has been published as part of *BMC Proceedings *Volume 8 Supplement 2, 2014: Proceedings of the 3rd Annual Symposium on Biological Data Visualization: Data Analysis and Redesign Contests. The full contents of the supplement are available online at http://www.biomedcentral.com/bmcproc/supplements/8/S2
